# The HeMoVal study protocol: a prospective international multicenter cohort study to validate cerebrospinal fluid hemoglobin as a monitoring biomarker for aneurysmal subarachnoid hemorrhage related secondary brain injury

**DOI:** 10.1186/s12883-022-02789-w

**Published:** 2022-07-18

**Authors:** Kevin Akeret, Raphael M. Buzzi, Moritz Saxenhofer, Kathrin Bieri, Deborah Chiavi, Bart R. Thomson, Manuela Grüttner-Durmaz, Nina Schwendinger, Rok Humar, Luca Regli, Tristan P. C. van Doormaal, Ulrike Held, Emanuela Keller, Michael Hugelshofer, Dominik J. Schaer, HeMoVal Research Group, Adrian Zuercher, Alexandra Grob, Amr Abdulazim, Basil Grüter, Constantin Roder, Danielle Wirz, Elisa Colombo, Gerrit A. Schubert, Isabelle Hostettler, Joshua Hägler, Nima Etminan, Muriel Helmers, Oliver Bozinov, Sophie Wang, Thomas Gentinetta, Vincens Kälin

**Affiliations:** 1 Department of Neurosurgery, Clinical Neuroscience Center, Universitätsspital and University of Zurich, Zurich, Switzerland; 2 Division of Internal Medicine, Universitätsspital and University of Zurich, Zurich, Switzerland; 3 CSL Behring AG, Research Europe, Berne, Switzerland; 4 Clinical Trials Unit (CTU), Berne, Switzerland; 5 Department of Biostatistics at Epidemiology, Biostatistics and Prevention Institute, University of Zurich, Zurich, Switzerland; 6 Clinical Trials Center – Research Ward (CTC-RW), University Hospital Zurich, Zurich, Switzerland; 7 Department of Neurology and Neurosurgery, University Medical Center, Utrecht, The Netherlands; 8 Neurointensive Care Unit, Department of Neurosurgery and Institute of Intensive Care Medicine, Universitätsspital and University of Zurich, Zurich, Switzerland

**Keywords:** Angiographic vasospasm, Biomarker, Delayed cerebral ischemia, Delayed ischemic neurological deficits, Haptoglobin

## Abstract

**Introduction:**

Preclinical studies provided a strong rationale for a pathophysiological link between cell-free hemoglobin in the cerebrospinal fluid (CSF-Hb) and secondary brain injury after subarachnoid hemorrhage (SAH-SBI). In a single-center prospective observational clinical study, external ventricular drain (EVD) based CSF-Hb proved to be a promising biomarker to monitor for SAH-SBI. The primary objective of the HeMoVal study is to prospectively validate the association between EVD based CSF-Hb and SAH-SBI during the first 14 days post-SAH. Secondary objectives include the assessment of the discrimination ability of EVD based CSF-Hb for SAH-SBI and the definition of a clinically relevant range of EVD based CSF-Hb toxicity. In addition, lumbar drain (LD) based CSF-Hb will be assessed for its association with and discrimination ability for SAH-SBI.

**Methods:**

HeMoVal is a prospective international multicenter observational cohort study. Adult patients admitted with aneurysmal subarachnoid hemorrhage (aSAH) are eligible. While all patients with aSAH are included, we target a sample size of 250 patients with EVD within the first 14 day after aSAH. Epidemiologic and disease-specific baseline measures are assessed at the time of study inclusion. In patients with EVD or LD, each day during the first 14 days post-SAH, 2 ml of CSF will be sampled in the morning, followed by assessment of the patients for SAH-SBI, co-interventions, and complications in the afternoon. After 3 months, a clinical follow-up will be performed. For statistical analysis, the cohort will be stratified into an EVD, LD and full cohort. The primary analysis will quantify the strength of association between EVD based CSF-Hb and SAH-SBI in the EVD cohort based on a generalized additive model. Secondary analyses include the strength of association between LD based CSF-Hb and SAH-SBI in the LD cohort based on a generalized additive model, as well as the discrimination ability of CSF-Hb for SAH-SBI based on receiver operating characteristic (ROC) analyses.

**Discussion:**

We hypothesize that this study will validate the value of CSF-Hb as a biomarker to monitor for SAH-SBI. In addition, the results of this study will provide the potential base to define an intervention threshold for future studies targeting CSF-Hb toxicity after aSAH.

**Study registration:**

ClinicalTrials.gov Identifier NCT04998370. Date of registration: August 10, 2021.

## Background

After an aneurysmal subarachnoid hemorrhage (aSAH) up to two-thirds of patients develop aSAH-related secondary brain injury (SAH-SBI), which negatively influences clinical outcome [1]. SAH-SBI typically occurs between day 4 and 14 after aneurysm rupture and manifests as angiographic vasospasms of large cerebral arteries (aVSP) [2], delayed cerebral ischemia (DCI) with radiologic demarcation of ischemic brain areas, or clinically diagnosed delayed ischemic neurologic deficits (DIND) [3]. There is an unmet need for early identification of patients at high risk for SAH-SBI. Clinical scores [4, 5], radiological scores [6–8], and day-to-day assessment with transcranial doppler sonography [9, 10] are widely used in a clinical setting, despite showing a limited accuracy [11]. Additionally, there is no clinically established biomarker to reliably monitor for SAH-SBI.

**Fig. 1:**
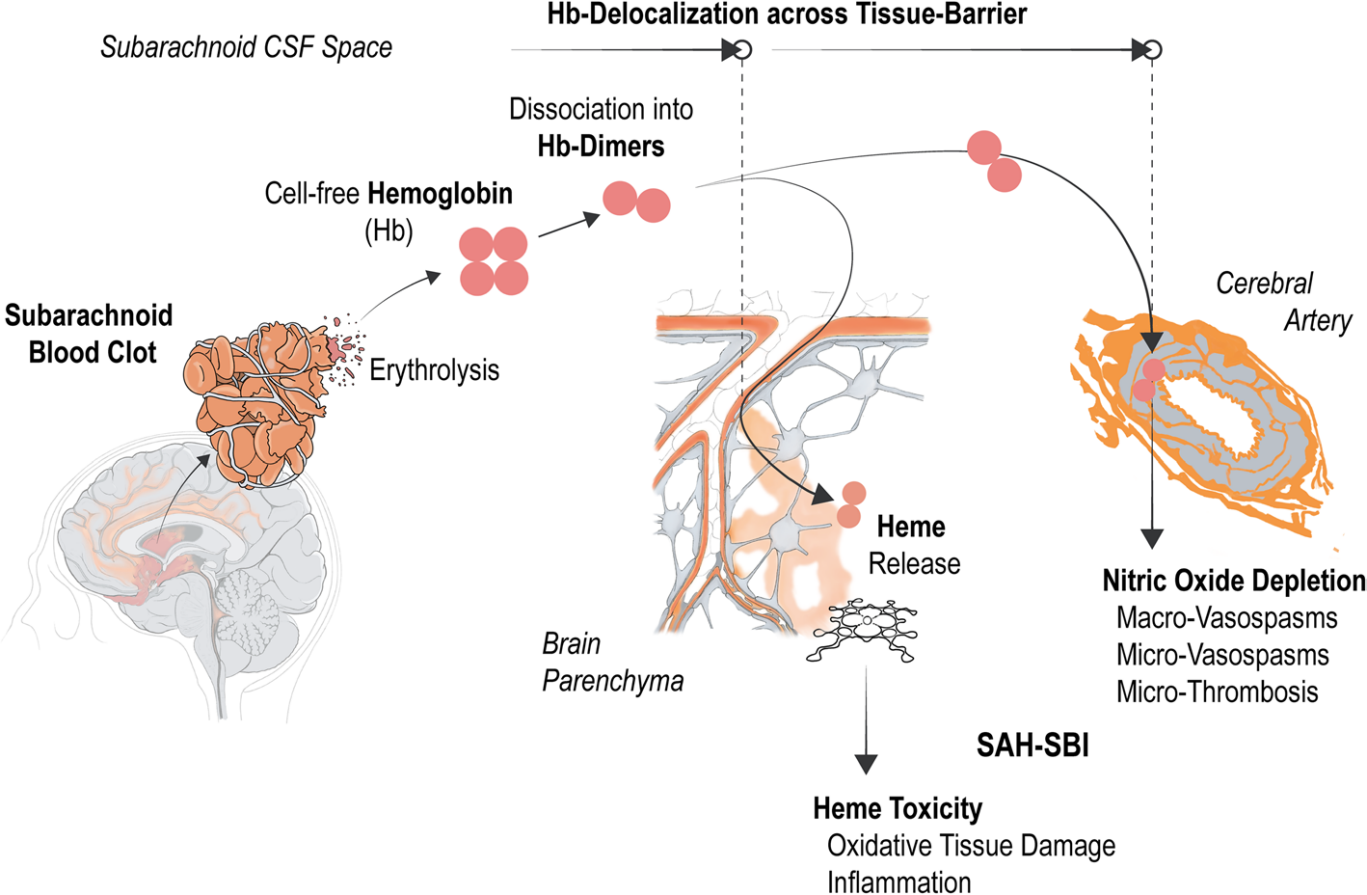
The pathophysiological concept of cell-free hemoglobin toxicity after aneurysmal subarachnoid hemorrhage: Usually with a delay of a few days after aneurysm rupture, hemoglobin (Hb) tetramers are released from lysing erythrocytes of the subarachnoid hematoma. The dimerization of Hb and hence the smaller molecular size allows for a delocalization across tissue barriers into vulnerable anatomical sites, such as the wall of blood vessels or the brain parenchyma. Within these compartments Hb exerts its toxicity through nitric oxide depletion and heme toxicity. (Illustrations by Rok Humar)

The pathophysiology of SAH-SBI is believed to be multifactorial involving macro- and microvascular dysfunction, neuroinflammation, neuronal apoptosis, and pathological electrical activity of the brain [1]. After the rupture of an intracranial aneurysm, blood components extravasate and disseminate into the cerebrospinal fluid (CSF), forming a subarachnoid hematoma. The subsequent decomposition of this hematoma, which is accompanied by erythrocytolysis, liberates cell-free hemoglobin into the CSF (CSF-Hb) [12]. CSF-Hb has been shown to reflect specific features of SAH-SBI through nitric oxide depletion and oxidative processes after heme liberation in ex vivo assays and translational in vivo models. Therefore, CSF-Hb is suspected of being one principal driver of the pathophysiological processes underlying SAH-SBI (Fig. 1) [12–14].

In a single center observational study, we found strong evidence for a positive association between daily measured EVD based CSF-Hb and the occurrence of SAH-SBI [12]. EVD based CSF-Hb showed a discrimination ability for SAH-SBI in general (AUC: 0.89 [0.85–0.92]), but also for the 3 clinical subgroups aVSP (AUC: 0.89 [0.82–0.93]), DCI (AUC: 0.88 [0.81–0.93]) and DIND (AUC: 0.89 [0.84–0.92]) that considerably exceeded the accuracy of established methods [12]. Receiver operating characteristic (ROC) analyses for SAH-SBI, aVSP, DCI, and DIND also generated preliminary insights into the clinically relevant toxicity range of EVD based CSF-Hb [12].

The strong underlying pathophysiological rationale, the preliminary clinical evidence for a positive association and high discrimination ability, and its easy measurability, position CSF-Hb as an attractive biomarker to monitor for SAH-SBI. The primary objective of this prospective international multicenter cohort study is to validate the positive association between EVD based CSF-Hb and SAH-SBI during the first 14 days post-SAH. Secondary objectives include the assessment of the discrimination ability of EVD based CSF-Hb for SAH-SBI and the definition of a clinically relevant range of EVD based CSF-Hb toxicity. Also, LD based CSF-Hb will be assessed for its association with and discrimination ability for SAH-SBI.

## Methods/design

### General study design and population

The HeMoVal study is designed as a prospective international multicenter observational study. The study population consists of patients admitted to a tertiary care center due to an aneurysmal subarachnoid hemorrhage.

#### Eligibility criteria

Participants fulfilling all of the following *inclusion* criteria are eligible for the study:

a. Age ≥ 18 years.

b. Hospital admission due to an aneurysmal subarachnoid hemorrhage (diagnosis radiologically confirmed)

The presence of any of the following *exclusion* criteria will lead to exclusion of the participant:

c. Non-aneurysmal subarachnoid hemorrhage (eg. trauma, perimesencephalic subarachnoid hemorrhage).
d. Participation in another study with CSF sampling or an interventional medical product within the 30 days preceding and during the present study.
e. Previous enrollment into the current study.

#### Enrollment

Patients with SAH admitted to one of the study centers will be screened for study inclusion as soon as possible after hospital admission (usually within 24 hours) by a member of the clinical study team at the respective center. The screening will be based on the initial CT imaging of the head to confirm SAH and vascular imaging (CT-Angiography, MR-Angiography, Digital Subtraction Angiography) for detection of a ruptured aneurysm. In patients fulfilling the inclusion and not meeting any of the exclusion criteria, informed consent will be obtained as required per local regulations.

#### Withdrawal and discontinuation

A study participant who discontinues study participation prematurely for any reason is defined as dropout. This may happen when:

- a patient who was incapable of giving informed consent at the time of inclusion does not agree to participate in the study once she/he is capable of giving consent. Any samples collected until the participant’s study withdrawal will be destroyed. Any data collected until the participant’s study withdrawal will be destroyed or, if destruction is not possible as in the study database, de-identified by removal of the patient ID. De-identified data will not be included in the analysis
- a patient who was incapable of giving informed consent at the time of inclusion and who remains permanently unable to judge and his/her legal representative does not agree to study participation Any samples collected until the participant’s study withdrawal will be destroyed. Any data collected until the participant’s study withdrawal will be destroyed or, if destruction is not possible as in the study database, de-identified by removal of the patient ID. De-identified data will not be included in the analysis
- a patient withdraws his/her study participation. Any samples and data collected until study withdrawal will remain coded for the analysis. It is not possible to anonymize the data and samples upon withdrawal as this would involve disproportionate effort.

### Study objectives

#### Primary objective

The primary objective of this study is to evaluate the strength of association between EVD based CSF-Hb (measured in the morning) and SAH-SBI (assessed in the afternoon of the same day) during the first 14 days post-SAH.

#### Secondary objectives

The secondary objectives are to investigate:

- Temporal profiles and dependence of EVD and lumbar drain (LD) based CSF-Hb:
  i. The temporal profile (day 1 to day 14 post-SAH) of EVD based CSF-Hb in the EVD cohort.
  ii. The temporal profile (day 1 to day 14 post-SAH) of LD based CSF-Hb in the LD cohort.
  iii. Difference (per day and overall) between EVD and LD based CSF-Hb from day 1 to day 14 post-SAH.
  iv. The strength of association between EVD based CSF-Hb (measured in the morning of day 1 to 14 post-SAH) and aneurysm location (assessed at study inclusion), hematoma volume (assessed at study inclusion), presence of intraventricular hemorrhage (assessed at study inclusion) and day post-SAH in the EVD cohort.
  v. The strength of association between LD based CSF-Hb (measured in the morning of day 1 to 14 post-SAH) and aneurysm location (assessed at study inclusion), hematoma volume (assessed at study inclusion), presence of intraventricular hemorrhage (assessed at study inclusion) and day post-SAH in the LD cohort.
- EVD based CSF-Hb and SAH-SBI, aVSP, DCI, and DIND in the EVD cohort:
  i. The temporal profiles (day 1 to day 14 post-SAH) of SAH-SBI, aVSP, DCI, and DIND in the EVD cohort.
  ii. The temporal profiles (day 1 to day 14 post-SAH) of EVD based CSF-Hb stratified by the presence or absence of SAH-SBI, aVSP, DCI, or DIND in the EVD cohort.
  iii. The strength of association between EVD based CSF-Hb (measured in the morning) and aVSP, DCI, and DIND (assessed in the afternoon of the same day) during the first 14 days post-SAH in the EVD cohort.
  iv. The discrimination ability of EVD based CSF-Hb (measured in the morning) for SAH-SBI, aVSP, DCI, and DIND (assessed in the afternoon of the same day) during the first 14 days post-SAH in the EVD cohort.
  v. The discrimination ability of EVD based CSF-Hb (measured in the morning) for the first occurence of SAH-SBI, aVSP, DCI, and DIND (assessed in the afternoon of the same day) during the first 14 days post-SAH in the EVD cohort.
  vi. The discrimination ability of EVD based CSF-Hb (measured in the morning of day X) for the first occurence of SAH-SBI, aVSP, DCI, and DIND (assessed in the afternoon of day X + 1) during the first 14 days post-SAH in the EVD cohort.
  vii. The discrimination ability of EVD based CSF-Hb (measured in the morning) for SAH-SBI, aVSP, DCI, and DIND (assessed in the afternoon of the same day) during day 4 and day 14 post-SAH in the EVD cohort.
- LD based CSF-Hb and SAH-SBI, aVSP, DCI, and DIND in the LD cohort:
  i. The temporal profiles (day 1 to day 14 post-SAH) of SAH-SBI, aVSP, DCI, and DIND in the LD cohort.
  ii. The temporal profiles (day 1 to day 14 post-SAH) of LD based CSF-Hb stratified by the presence or absence of SAH-SBI, aVSP, DCI, or DIND in the LD cohort.
  iii. The strength of association between LD based CSF-Hb (measured in the morning) and SAH-SBI, aVSP, DCI, and DIND (assessed in the afternoon of the same day) during the first 14 days post-SAH in the LD cohort.
  iv. The discrimination ability of LD based CSF-Hb (measured in the morning) for SAH-SBI, aVSP, DCI, and DIND (assessed in the afternoon of the same day) during the first 14 days post-SAH in the LD cohort.
  v. The discrimination ability of LD based CSF-Hb (measured in the morning) for the first occurence of SAH-SBI, aVSP, DCI, and DIND (assessed in the afternoon of the same day) during the first 14 days post-SAH in the LD cohort.
  vi. The discrimination ability of LD based CSF-Hb (measured in the morning of day X) for the first occurence of SAH-SBI, aVSP, DCI, and DIND (assessed in the afternoon of day X + 1) during the first 14 days post-SAH in the LD cohort.
  vii. The discrimination ability of LD based CSF-Hb (measured in the morning) for SAH-SBI, aVSP, DCI, and DIND (assessed in the afternoon of the same day) during day 4 and day 14 post-SAH in the LD cohort.
- EVD based CSF-Hb and 3-months follow-up status:
  i. The strength of association between EVD based CSF-Hb during the first 14 days post-SAH and chronic hydrocephalus at 12 weeks follow-up in the EVD cohort.
  ii. The strength of association between EVD based CSF-Hb during the first 14 days post-SAH and functional outcome (GOSE, mRS) at 12 weeks follow-up in the EVD cohort.
- LD based CSF-Hb and 3-months follow-up status:
  i. The strength of association between LD based CSF-Hb during the first 14 days post-SAH and chronic hydrocephalus at 12 weeks follow-up in the LD cohort.
  ii. The strength of association between LD based CSF-Hb during the first 14 days post-SAH and functional outcome (GOSE, mRS) at 12 weeks follow-up in the LD cohort.

### Study endpoints

Here we provide a summary of the endpoints assessed in this study. A detailed list with characterization of the variables can be found online (10.5281/zenodo.5556317).

#### CSF-Hb (biomarker)

CSF-Hb constitutes the biomarker assessed in this study. CSF-Hb levels (oxyhemoglobin) will be quantified in the CSF samples obtained from patients after aSAH using spectral deconvolution, as described previously [12, 13].

#### Primary endpoint

The primary endpoint of this study is the occurrence of SAH-SBI during the monitoring period. SAH-SBI is a categorical variable that will be derived as a composite from the categorical secondary endpoints aVSP, DCI, and DIND: If any one or more of the three secondary endpoints (aVSP, DCI, or DIND) is present on the corresponding day, SAH-SBI is considered to be present. If neither information on aVSP (no imaging performed), nor DCI (no imaging performed) or DIND (patient not clinically assessable) could be obtained, SAH-SBI is considered to be non-assessable. In any other instance, SAH-SBI is considered not to be present.

#### Secondary endpoints

Secondary endpoints, which will be assessed during the first 14 days post-SAH on a daily basis, are: angiographic vasospasms (aVSP), delayed cerebral ischemia (DCI), delayed ischemic neurologic deficits (DIND), elements of triple H therapy, spasmolysis, surgical decompression.

The definition of aVSP comprises a narrowing of cerebral arteries based on a DSA, CTA or MRA. In the absence of an appropriate imaging procedure on the respective day, this will be noted as *no imaging performed*. Categorical variable [present, absent or no imaging performed].

DCI is defined as new perfusion deficit or new infarction in native and/or contrast-enhanced CT/MRI. In the absence of an appropriate imaging procedure on the respective day, this will be noted as *no imaging performed*. Categorical variable [present, absent or no imaging performed].

DIND is defined as a new focal neurological deficit or a decrease in GCS of at least 2 points for at least 2 hours. In case the patient cannot be clinically assessed (e.g., sedation), this will be noted as *non-assessable*). Categorical variable [present, absent or non-assessable].

Secondary endpoints, which will be assessed at the 12 weeks (+/− 2 weeks) follow-up: Chronic hydrocephalus, Glasgow Outcome Scale Extended (GOSE), modified Rankin Scale (mRS).

#### Safety endpoints

Safety endpoints, which will be assessed during the first 14 days post-SAH on a daily basis in the afternoon, always at approximately the same time: CSF infection, revision due to surgical site infection at the EVD skin entrance.

#### Demographic/baseline measures

Measures assessed at the time of study inclusion (usually within 24 hours of hospital admission): Patient demographics (age, sex), medical history (antithrombotics, diabetes, hypertension, coronary artery disease, smoking), clinical assessment (Glasgow Coma Scale (GCS), pupil status, cranial nerve deficit, focal neurological deficit, headache, neck stiffness), clinical SAH scores at admission (World Federation of Neurological Surgeons (WFNS), Hunt & Hess grade), Radiological features (aneurysm location, aneurysm size, intracerebral hemorrhage, intraventricular hemorrhage, hematoma volume), radiological SAH scores at admission (modified Fisher grade, Barrow Neurological Institute (BNI) grade), aneurysm therapy.

### Study assessments

**Fig. 2:**
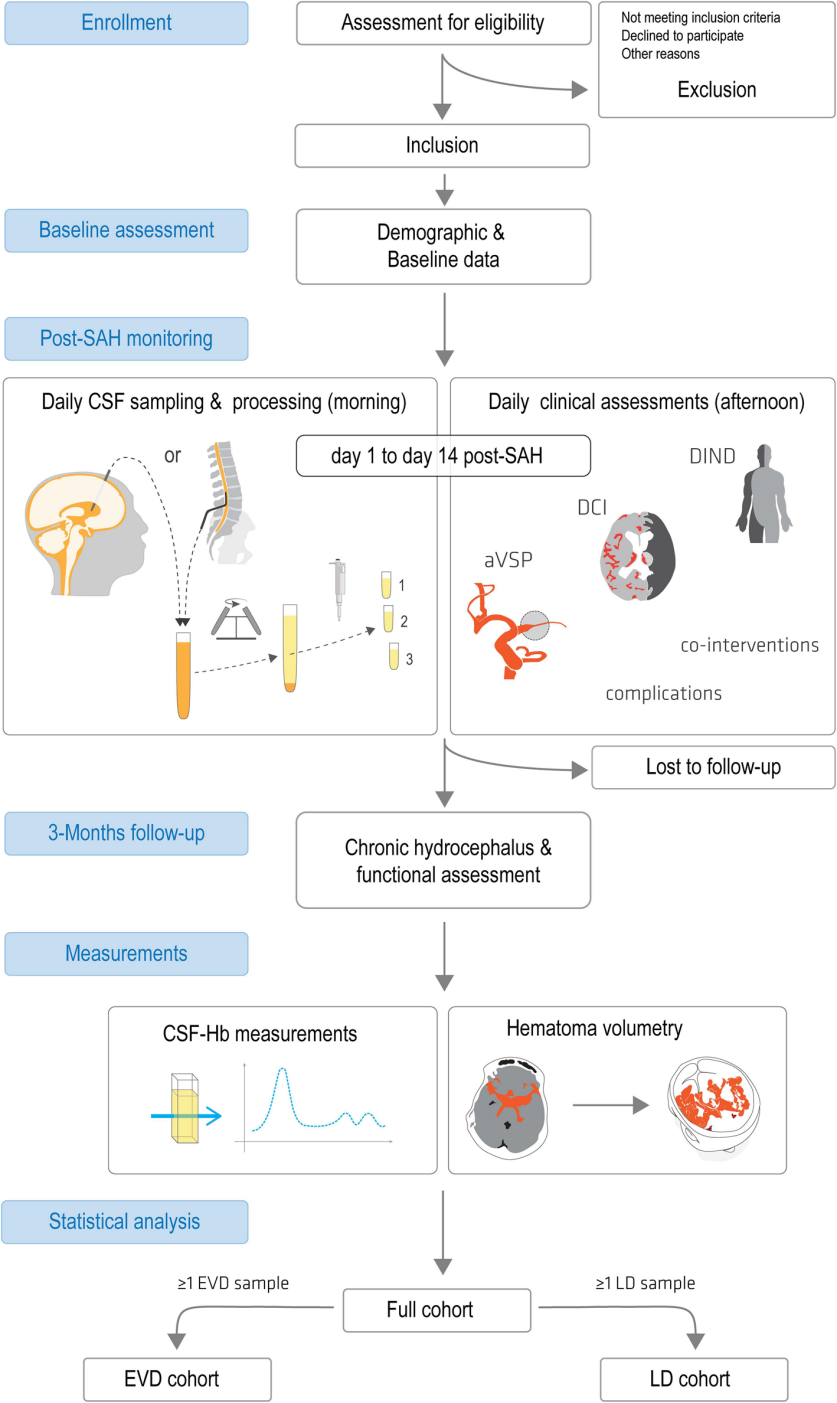
HeMoVal study assessment flowchart: Graphical representation of the different phases of assessment (enrollment, baseline assessment, monitoring, 3-months follow-up), measurements and analysis in the HeMoVal study

A detailed definition and description of each variable, including levels (for factors) and range (for continuous variables), and assessment procedure, can be found online (10.5281/zenodo.5556317). It is ensured that all members of the clinical study team will be specifically trained in the study-specific assessments. The assessments in this study are divided into *baseline assessment*, *post-SAH monitoring*, *3-months follow-up*, and *measurements*. Table 1 shows the assessment schedule, the study flowchart is given in Fig. 2.

**Table 1:** HeMoVal study assessment table. Overview of the different study periods (enrollment, baseline assessment, monitoring, 3-months follow-up, measurements) with the corresponding time schedule and assessments

Study periods	Enrollment	Baseline assessment	Monitoring	3-months follow-up	Measurements
**Time**	Hospital admission	Study inclusion	Daily after inclusion for max. 14 days postSAH	+ 12 weeks after event (+/− 2 weeks)	N/A
**Eligibility Criteria**	x				
**Patient information & consenting**	x				
**Demographic/baseline measures**		x			
**CSF-sampling**			x (daily, if EVD/LD)		
**aVSP**			x (daily, if imaging)		
**DCI**			x (daily, if imaging)		
**DIND**			x (daily, if assessable)		
**Spasmolysis**			x (daily)		
**Intracerebroventricular rtPA**			x (daily)		
**Triple H therapy**			x (daily)		
**Surgical decompression**			x (daily)		
**Nimodipine therapy**			x (daily)		
**CSF infection**			x (daily)		
**Surgical site infection**			x (daily)		
**Chronic hydrocephalus**				x	
**GOSE**				x	
**mRS**				x	
**Hematoma volume**					x
**CSF-Hb**					x

#### Baseline assessment

Gathering of demographic (age, sex) and baseline (medical history, clinical assessment, clinical SAH scores at admission, radiological features, aneurysm therapy) patient data. Performed directly after study inclusion by a trained member of the clinical study team.

#### Post-SAH monitoring

CSF sampling and clinical assessments will be performed on a daily basis between day 1 and day 14 post-SAH, always at approximately the same time of the day (CSF sampling in the morning, clinical assessment in the afternoon) by a trained member of the clinical study team.

CSF sampling will be performed via EVD or LD if present. The decision regarding the insertion of an EVD or LD is made on a clinical basis by the treating physician independently of this study. If an EVD or LD is present, 2 ml of CSF will be sampled daily, centrifuged at 1500 G for 15 minutes, and subsequently stored at − 80 °C.

The clinical assessment of patients for the primary and secondary endpoints will be blinded to CSF sampling, since the macroscopic aspect of CSF after centrifugation may provide an indication of the CSF-Hb level. The definition of aVSP comprises a narrowing of cerebral arteries based on a DSA, CTA or MRA. In the absence of an appropriate imaging procedure on the respective day, this will be noted as *no imaging performed*. DCI is defined as a new perfusion deficit or a new infarction in native and/or contrast-enhanced CT/MRI. In the absence of an appropriate imaging procedure on the respective day, this will be noted as *no imaging performed*. DIND is defined as a new focal neurological deficit or a decrease in GCS of at least 2 points for at least 2 hours. In case the patient cannot be clinically assessed (e.g., under sedation), this will be noted as *non-assessable*). Other secondary endpoints assessed include elements of triple H therapy, spasmolysis, and surgical decompression. CSF infection and revision due to surgical site infection at the EVD skin entrance comprise safety endpoints.

#### Follow-up

The assessment will be performed at the 12 weeks (+/− 2 weeks) follow-up visit. This will be done on site if possible. If this is not possible, a consultation by telephone will be conducted. The presence of chronic hydrocephalus is defined as the dependence on permanent CSF diversion which was not present before aneurysm rupture. The functional status scores will be assessed with the Glasgow Outcome Scale Extended (GOSE) and the modified Rankin Scale (mRS).

#### Measurements

CSF-Hb measurements and hematoma volumetry will be performed after completion of the monitoring period of the last included patient in this study.

CSF-Hb measurements: CSF-Hb measurements will be performed centralized in the Preclinical Research in Internal Medicine (PRIME) Laboratory, Schlieren, Switzerland. Absorption spectra in the visual range between 350 and 650 nm will be measured and quantification of the oxyhemoglobin in CSF will be performed using spectral deconvolution, as described previously [12, 13]. CSF-Hb measurements are performed by a person blinded to the patients demographic/baseline measures and their clinical assessment/endpoints.

Hematoma volume: Assessed on the initial CT scan of each patient. The CT scan will be uploaded by the local investigators in coded form (identifying information replaced by patient ID) to a HIPAA compliant platform (Lumi, Augmedit bv, Naarden, the Netherlands), from where automated centralized quantification of hematoma volume is performed. The quantification of the hematoma volume is performed by a person blinded to the patient’s CSF sampling/CSF-Hb values and their clinical assessment/endpoints.

#### Serious events

A serious event is defined as any adverse event where it cannot be excluded, that the event is attributable to the sampling of biological material or the collection of health-related personal data, and which:

- requires inpatient treatment not envisaged in the protocol or extends a current hospital stay,
- results in permanent or significant incapacity or disability, or
- is life-threatening or results in death.

If a serious event occurs, the research project will be interrupted. The responsible Ethics Committee(s) and Competent Authority will be notified according to the locally relevant regulations, if required.

### Data management and quality assurance

#### Quality measures

For quality assurance representatives of the responsible Ethics Committee may visit the research sites. Direct access to the source data and all project related files and documents will be granted on such occasions. Central and on-site monitoring (CTU Berne, Switzerland) will be part of the quality control activities implemented for this study. During the study, the central data monitor will check the eCRFs on a weekly basis and lock complete visits. All involved parties will keep participant data strictly confidential.

#### Data recording and source data

The CRFs in this trial are implemented electronically using a dedicated electronic data capturing (EDC) system (REDCap, https://www.project-redcap.org/). The EDC system was activated for the study after successfully passing a formal test procedure. All data entered in the CRFs are stored on a Linux server in a dedicated mySQL database. Responsibility for hosting the EDC system and the database lies with CTU Berne. The initial CT scan of each patient will be uploaded and stored for the duration of the study in coded form (identifying information replaced by patient ID) on the HIPAA compliant platform *Augsafe*. The data from the CSF-Hb analysis are recorded in the EDC system of the Preclinical Research in Internal Medicine (PRIME) Laboratory, Schlieren, Switzerland. For the statistical analysis, the data will be merged to an export of the eCRF, based on the patient ID. Source data will be available at each study site in the respective patient files, either electronically or on paper. The source data will include the original documents relating to the study, as well as the medical treatment and medical history of the patients. In the Investigator Site File and Trial Master File the informed consent forms and other relevant study-specific documents will be recorded. Any patient files and source data will be archived for the longest possible period of time according to the feasibility of the investigational site.

#### Confidentiality and coding

The server hosting the EDC system and the database is kept in a locked server-room. Only the system administrators have direct access to the server and back-up tapes. A role concept with personal passwords (site investigator, statistician, monitor, administrator etc.) regulates permission for each user to use the system and database as he/she requires. Authorized to enter data into the eCRF are the local trial team staff according to the database access list. The local project leader is responsible for proper training and instruction of the trial personnel filling data into the eCRF. Study-related data of the patient will be collected in a coded manner. The names of the patients will not be disclosed. A code (unique, consecutively numbered per center) will be attributed to each patient registered. All data entered into the CRFs are transferred to the database using Transport Layer Security (TLS) encryption. Each data point has attributes attached to it identifying the user who entered it with the exact time and date. Retrospective alterations of data in the database are recorded in an audit table. Time, table, data field and altered value, and the person are recorded (audit trail). A multi-level back-up system is implemented. Back-ups of the whole system including the database are run internally several times per day and on external harddrives once a day. The back-up tapes are stored in a secure place in a different building.

#### Biological material

Biological material in this project will be coded using a barcode system that will be linked to the patient ID and sampling attributes. Biological material is appropriately stored in a restricted area only accessible to authorized personnel. CSF samples from local sites will be shipped to Research Biobanking Service Center (RBSC), University Hospital Zurich, Zurich, Switzerland.

#### Retention and destruction of study data and biological material

At final analyses, data files will be extracted from the database into the statistical software R to be analyzed. After database lock, the status of the database is recorded in special archive tables. The sponsor will keep the Trial Master File, the extracted data, the meta data and final reports for at least 10 years. Each site will archive its study documents for at least 10 years. Leftover CSF samples will be stored for a maximum time period of 10 years. At the end of this period they will be destroyed according to local law and regulations.

### Sample size

The primary objective of the study focuses on patients included with EVD (EVD cohort) while secondary objectives also consider patients with LD (LD cohort) and patients irrespective of any drainage system (full cohort). The sample size estimation is based on an anticipated effect size of 0.1, a power of 90% and accounts for between-site variability using a generalized linear mixed model. This calculation results in a minimum of 6 participating study sites and 30 patients per site with an EVD. Considering an anticipated drop-out rate of 15% and heterogenous recruitment rates at the sites, we plan to include 8 sites and a maximal study population of 250 patients with EVD. Based on our clinical experience, we assume that only every second subject receives an EVD. Thus, we assume to include a maximum of 500 subjects. The recruitment will be stopped as soon as 250 patients with EVD are included.

### Statistical analysis

Statistical analyses will be performed in R in an R studio environment on a Mac computer operating system [15]. The entire statistical code will be written by two authors (KA and RMB). The reviewing statistician (UH) will have an overview of the entire code. A second review statistician will independently reproduce the primary analyses. No interim analysis will be performed. The final analysis will be conducted after completion of the study, i.e. when every patient has reached the 12 weeks follow-up, and data has been cleaned by the central data monitor. For analysis, the study population will be divided into the following three populations: EVD cohort (at least one CSF-sampling via an EVD), LD cohort (at least one CSF-sampling via an LD), full cohort (all included patients, irrespective of their CSF-sampling status). The null hypothesis is that there is no association between the concentration of EVD based CSF-Hb and the occurrence of SAH-SBI during the first 14 days post-SAH in the EVD cohort. The confirmatory alternative hypothesis is that there is an association between the two. The primary analysis evaluates the strength of association between SAH-SBI (dependent binary variable) and EVD based CSF-Hb (independent continuous variable) in the EVD cohort based on a generalized additive model (GAM) with day post-SAH as continuous covariable (non-linear spline-fit with 4 knots), and repeated observations in the same patients over time and each study site accounted for with random intercepts. The likelihood ratio test *p*-value will be used to test the null hypothesis of no effect of the biomarker at a 5% significance level. In the primary analysis, no adjustment of the significance level for multiple testing is needed. Detailed description on summary statistics, secondary and safety endpoint analyses can be found online in the statistical analysis plan (10.5281/zenodo.5556317). For secondary analyses, no level of statistical significance will be defined. Instead, the results of secondary analyses will be interpreted based on the level of evidence (*p* < 0.001: very strong evidence; *p* < 0.01: strong evidence; *p* < 0.05 evidence; *p* < 0.1 weak evidence; *p* > 0.1: no evidence [16]) and confidence intervals will be provided. No confirmatory claims will be based on the secondary analyses. The results of the study will be reported according to STROBE guidelines for reporting of observational research [17].

### Ethics and dissemination

The HeMoVal study is conducted in accordance with the Declaration of Helsinki and national and international standards of ICH-GCP E6(R2). Potential participants or their legal representatives receive detailed written and oral information on the study procedures and all participants or their legal representatives provide written informed consent. The study protocol will be approved by the local ethics committees of all participating study sites. To date, the study protocol has been approved by the ethics committee of the Canton of Zurich, Switzerland (KEK 2021–01023) for Zurich (CH), Aarau (CH), and St. Gallen (CH). The primary and secondary results of this study will be published in peer-reviewed journals. Authors of these publications are persons who planned and/or conducted the study and/or did parts of the statistical analysis.

### Study registration

ClinicalTrials.gov Identifier NCT04998370. Date of registration: August 10, 2021.

### Current status of the study

Recruitment for the HeMoVal study is currently starting at multiple centers across Switzerland, Germany and the Netherlands. At the University Hospital of Zurich (Switzerland) the recruitment started on August 15, 2021, and at the Kantonsspital Aarau (Switzerland) on August 25, 2021. At the time of submission of this protocol, 21 patients have been enrolled in the HeMoVal study.

## Discussion

Preclinical studies provide a strong rationale for a causal pathophysiological link between CSF-Hb and SAH-SBI [12–14]. Also, CSF-Hb demonstrated to be a promising clinical biomarker to monitor for SAH-SBI in a prospective single-center observational study [12]. The primary objective of the herein described prospective international multicenter observational cohort study is to externally validate the positive association between EVD based CSF-Hb and SAH-SBI during the first 14 days post-SAH. Secondary objectives include the assessment of the discrimination ability of EVD based CSF-Hb for SAH-SBI and the definition of a clinically relevant range of EVD based CSF-Hb toxicity. Additionally, LD-based CSF-Hb will be assessed for its association with and discriminatory ability for SAH-SBI.

There is considerable heterogeneity in the use of the terms aVSP, DCI and DIND in both literature and clinical practice [18]. The composite outcome SAH-SBI constitutes the primary endpoint of the HeMoVal study, conceptualizing the pathophysiological and clinical interconnection between aVSP, DCI and DIND, thus increasing statistical power. The concept of SAH-SBI is strengthened by the previous finding of a dose-dependent relation between EVD based CSF-Hb and aVSP, DCI and DIND with increasing toxicity ranges [12]. As secondary endpoints, aVSP, DCI and DIND will be assessed individually. The strictly delineated definition of these endpoints based on angiographic vessel narrowing (aVSP), perfusion deficit/infarction (DCI) or clinical deterioration (DIND) empowers conclusions regarding CSF-Hb toxicity range and intervention thresholds.

The HeMoVal study will enroll all patients who are diagnosed with aSAH at admission, thus ensuring that start of CSF sampling from a newly introduced EVD or LD is possible at each day of the 14-day monitoring period. For the statistical analysis, the cohort will be stratified into an EVD cohort (at least one CSF sample obtained via an EVD), LD cohort (at least one CSF sample obtained via an LD), and a full cohort (all patients admitted with an aSAH). Distinguishing between EVD and LD cohorts anticipates potential differences in the temporal profiles of EVD and LD based CSF-Hb, and hence their association to and discrimination ability for SAH-SBI. Despite the anatomical communication between the intraventricular and lumbar CSF spaces, substantial differences in the protein levels of samples obtained from the two compartments were described in patients with multiple sclerosis [19]. The full cohort serves as reference for the generalizability of the finding in the EVD and LD cohort to the overall aSAH population.

The strength of association between CSF-Hb and SAH-SBI, aVSP, DCI, and DIND will be quantified using a GAM. GAMs blend properties of generalized linear models with additive models [20]. The data structure of the HeMoVal study is characterized by repeated CSF-Hb measurements and clinical assessments (aVSP, DCI, DIND, and hence the composite outcome SAH-SBI) in the same subjects during the 14 day monitoring period, for which we will adjust with a random intercept. In the same way, there will be adjustment for potential intracluster correlation per study site. In addition, based on the results of our pilot study, we expect a non-linear relation of SAH-SBI, aVSP, DCI, and DIND to the day post-SAH with a peak between post-SAH day 8 and 12 [12]. A GAM provides the flexibility to incorporate these properties, including smooth functions of covariates, while maintaining some degree of explainability [20].

The collection of CSF samples for the HeMoVal study is a manipulation at the closed EVD/LD drainage system, which may raise concerns about an increased risk for CSF infections. Reported infection rates of EVD/LD systems in clinical routine range from 0 to 32%. However, rates around 10% are usually described [21–23]. Since the introduction of antimicrobial-coated catheters, infection rates of less than 2% have been reported at many centers [24–29]. The single most important risk factor for CSF infection in patients with EVD/LD is the duration of external CSF diversion [21, 23, 30–32]. This factor is not influenced by the HeMoVal study, since the indications for insertion and removal of CSF diversion devices are made independent of the study exclusively based on clinical criteria assessed by the independent treating physician (e.g., increased intracranial pressure, need for pressure monitoring). The HeMoVal study increases the number of manipulations of the EVD/LD system compared to clinical routine due to the daily sampling regimen. Evidence regarding the existence, strength, and causality of an association between such manipulations and the incidence of CSF infections is sparse and inconclusive [21, 23, 30–39], but theoretically an increased risk of infection with each manipulation must be assumed. However, strict adherence to aseptic techniques limits this risk to an absolute minimum [40–42]. In a prospective single center observational study involving 47 patients, adhering to the same protocol as the HeMoVal study, only one patient (2.1%) developed CSF infection [12]. This lies in the lower range of recent reports on the incidence of CSF infections in patients with EVD/LD [24–29]. We therefore consider the additional risk of CSF infection due to repetitive sampling under aseptic measures in this study to be minimal. However, CSF infection is included as a safety endpoint in the assessment during the monitoring period*.* Further, we don’t expect an increased risk of symptomatic CSF overdrainage due to the sampling regimen in the HeMoVal study, since the collected 2 ml of CSF per day represent only a fraction of the regular hourly drainage volume needed for the treatment of posthemorrhagic hydrocephalus [22] and because the total daily drainage volume remains unchanged. Overall, we consider that the potential scientific insights and clinical implications of the HeMoVal study clearly outweigh its risk.

In this study, a timed imaging protocol was deliberately avoided since it would have had to be performed at high frequency due to the variability of the temporal occurrence of aVSP and DCI and would therefore have been associated with an unacceptable additional burden for the patients (e.g., radiation exposure during CTA, transport risk of intubated patients). Moreover, it would not have been a compelling substitute for clinically indicated imaging in the setting of neurologic deterioration. Thus, in the HeMoVal study, we follow the real clinical situation with imaging according to clinical indication, which increases the variability of the results, but also their generalizability.

The HeMoVal study has the potential to strengthen the evidence for a positive association between CSF-Hb levels and the occurrence of SAH-SBI. In the future, CSF-Hb may serve as a biomarker to monitor for SAH-SBI. CSF processing and photospectrometry based measurements constitute easy to conduct procedures, which could be readily implemented as point-of-care test. The HeMoVal study is intended to provide generalizable data on the discrimination ability of CSF-Hb for SAH-SBI and thus to provide the basis to define an intervention threshold. Final choice on a clinical intervention threshold will additionally depend on the individual burden and the economic costs associated with false-positive and false-negative results.

Given the hypothesized causal pathophysiological link, CSF-Hb also represents a potential drug target. Targeting CSF-Hb through intrathecal application of the Hb-scavenger haptoglobin offers both preventive and therapeutic potential [12, 13]. By complexation, haptoglobin was shown to compartmentalize cell-free Hb in the CSF space, thereby inhibiting Hb delocalization into the brain parenchyma or smooth muscle cell layer of cerebral arteries [13]. Both ex vivo and in vivo, haptoglobin exhibited an anti-vasospastic effect on cerebral arteries [12, 13]. Haptoglobin also protected from lipid-peroxidation in an ex vivo assay for quantification of the oxidative potential [12]. The HeMoVal study will provide more generalizable information on the clinically relevant toxicity range of EVD and LD based CSF-Hb through ROC analyses for SAH-SBI, aVSP, DCI, and DIND. Those results are expected to inform the development of haptoglobin as a CSF-Hb targeting therapeutic in patients after aSAH.

## Data Availability

Depending on the type of data and associated privacy regulations, data from the HeMoVal study will be made publicly available via the corresponding author, upon reasonable request.

## Abbreviations

aSAH: Aneurysmal subarachnoid hemorrhage
AUC: Area under the curve
aVSP: Angiographic vasospasm
BNI: Barrow Neurological Institute
CSF: Cerebrospinal fluid
CSF-Hb: Hb in CSF of patients after aSAH
DCI: Delayed cerebral ischemia
DIND: Delayed ischemic neurological deficits
EVD: External ventricular drain
GAM: Generalized additive model
GCS: Glascow Coma Scale
GOSE: Glascow Outcome Scale - Extended
ICH-GCP: International Council for Harmonisation - Good Clinical Practice
IVH: Intraventricular hemorrhage
LD: Lumbar drain
mRS: Modified Rankin Scale
ROC: Receiver operating characteristic
SAH-SBI: subarachnoid hemorrhage related secondary brain injury;
WFNS: World Federation of Neurosurgical Societies
